# Chronic musculoskeletal pain, catastrophizing, and physical function in adult women were improved after 3-month aerobic-resistance circuit training

**DOI:** 10.1038/s41598-021-91731-0

**Published:** 2021-07-22

**Authors:** Seira Sato, Sho Ukimoto, Takashi Kanamoto, Nao Sasaki, Takao Hashimoto, Hikaru Saito, Eisuke Hida, Tomoharu Sato, Tatsuo Mae, Ken Nakata

**Affiliations:** 1 Medicine for Sports and Performing Arts, Department of Health and Sport Sciences, Osaka University Graduate School of Medicine, 2-2 Yamadaoka, Suita, Osaka 565-0871 Japan; 2 2 Japan Drug Development and Medical Affairs, Eli Lilly Japan K.K., 5-1-28 Isogamidori Chuo-ku, Kobe, Hyogo 651-0086 Japan; 3 Curves Japan Co., Ltd., 3-9-1 Shibaura, Minato-ku, Tokyo, 108-0023 Japan; 4 Department of Biostatistics and Data Science, Osaka University Graduate School of Medicine, 2-2 Yamadaoka, Suita, Osaka 565-0871 Japan; 5 Department of Sports Medical Biomechanics, Osaka University Graduate School of Medicine, 2-2 Yamadaoka, Suita, Osaka 565-0871 Japan

**Keywords:** Rehabilitation, Health care economics, Health care, Disease prevention

## Abstract

Although exercise is beneficial for chronic musculoskeletal pain (CMP), the optimal type and amount of exercise are unclear. This study aimed to determine the impact of circuit training that combines aerobic and resistance exercises on adult women with CMP. A total of 139 women with CMP underwent circuit training for 3 months and were asked to complete the following questionnaires at baseline and 3 months later: Numeric Rating Scale (NRS), Pain Catastrophizing Scale (PCS), Roland-Morris Disability Questionnaire (RDQ), Shoulder36, and Knee injury and Osteoarthritis Outcome Score (KOOS). Significant improvements were observed in NRS, PCS, RDQ, and KOOS activities of daily living (ADL) scores after the intervention relative to baseline (p < 0.0001, p = 0.0013, 0.0004, and 0.0295, respectively), whereas shoulder function did not improve. When considering the impact of exercise frequency, NRS scores improved regardless of exercise frequency. Furthermore, PCS, RDQ, and KOOS scores improved in participants who exercised at least twice a week (24 sessions over the course of 3 months). In conclusion, CMP, pain catastrophizing, and physical function in adult female fitness club participants with CMP of NRS 4 or higher improved after 3 months of aerobic-resistance circuit training.

## Introduction

Chronic musculoskeletal pain (CMP) is a multifactorial condition that negatively impacts not only physical function, but also mental functioning and quality of life^1^. The National Livelihood Survey in Japan reported that low back pain, shoulder stiffness, and joint pain are the most common symptoms experienced by the general Japanese population^2^. Approximately 20–30% of the adult population has chronic pain^3,4^ and the socio-demographic factors associated with chronic pain include female sex and older age^5^.

The pathology of CMP involves intricately related biological and psychosocial factors because it has been linked to numerous physical and mental conditions^4^. Changes in these factors resulting from CMP include muscle weakness associated with reduced daily activity, somnipathy, malnutrition, drug dependence, dependence on family, isolation from family or society, decline in job performance, and economic burden^6,7^. Pain conditions are reportedly responsible for 21% of years lived with disability (YLDs)^8^. CMP frequently occurs as a result of a disease or injury. However, it is not merely an accompanying symptom, but rather a separate condition in its own right, with its own medical definition and taxonomy^9^. In addition, pain catastrophizing is an important psychosocial predictor of the course and experience of chronic pain^10^, and it has been found to be inversely related to muscular endurance^11^. This tendency has been proven to negatively impact the neuromuscular, cardiovascular, immune, and neuroendocrine systems^12^. Therefore, research on CMP treatments, which not only relieve pain but also improve physical and psychosocial factors, is important^13^.

Exercise therapy, encompassing a wide range of interventions such as general (aerobic) exercise, specific body region exercises for strengthening and flexibility, continuing normal physical activities, and increasing general physical activity levels, is a core treatment option for patients with CMP^1^. Exercise therapy is particularly important given that existing pharmacological options for chronic pain are often limited by side effects, abuse potential, and overall efficacy^14^. There is little evidence supporting one particular type and amount of exercise for CMP^15^. Aerobic exercise has been shown to decrease pain sensitivity in patients with CMP^16^. However, a systematic review found that unlike in resistance training, aerobic exercise programs showed no effect in reducing chronic low back pain^17^. Previous studies have demonstrated that combination exercise training, which involves aerobic and strength exercise training, is likely to provide the individual effects unique to aerobic exercise and resistance training, respectively^18^. Combined aerobic and resistance training can improve lean body mass, muscular strength, body composition, and glycemic control in older adults^19–21^. Moreover, a meta-analysis showed that combination training has a greater effect on cognitive function in older adults than aerobic exercise training alone^22^. Circuit training is defined as the performance of 6–12 exercises in sequential order with little to no break between them^23^. A novel type of circuit training program involves a combination of the two aforementioned training modalities, namely, a resistance training set followed immediately by an aerobic exercise interval (a simultaneous aerobic-resistance training method). The effects of circuit training on several chronic disorders have been reported, and positive effects have been demonstrated on body composition, including reductions in body fat and body mass index (BMI)^24^. The aerobic-resistance circuit training program can elicit a greater cardiorespiratory response and similar muscular strength gains with less time commitment compared with a traditional resistance training program combined with aerobic exercise^25^. Moreover, this type of circuit training program was found to be feasible and safe in men with post-myocardial infarction^26^. The nature of circuit training may provide an effective, well-rounded exercise program to improve the overall fitness of older adults^21^. There have only been few reports on the effects of aerobic-resistance circuit training on pain. Circuit training has been reported to decrease postoperative pain after total knee arthroplasty^27^ and pain perception in women with fibromyalgia^28^. Although these studies focused on postoperative pain and pain perception, the effect of aerobic-resistance circuit training for reducing CMP is still unknown.

Recent evidence also suggests that older women are more likely to live with chronic pain than older men^9,29^. In addition to a female preponderance for chronic pain, women consistently report lower pain thresholds, lower pain tolerance, and greater unpleasantness with pain with different analgesic sensitivity^30^. Previous reports have indicated that there might be sex-specific mediators affecting the association of demographic variables with pain intensity and physical function, suggesting the need for a sex-specific approach when treating chronic pain^31^. This highlights that it is beneficial to focus on one sex only when examining the effects of exercise therapy, and since CMP is more common in women, there is a greater clinical need for treatment among women.

We hypothesized that aerobic-resistance circuit training would have positive effects on pain, pain catastrophizing, and physical function in women with CMP. To investigate this, the present questionnaire-based cohort study aimed to evaluate the effects of aerobic-resistance circuit training for 3 months in women with CMP who became new members of fitness facilities.

## Results

### Participant characteristics

**Figure 1:**
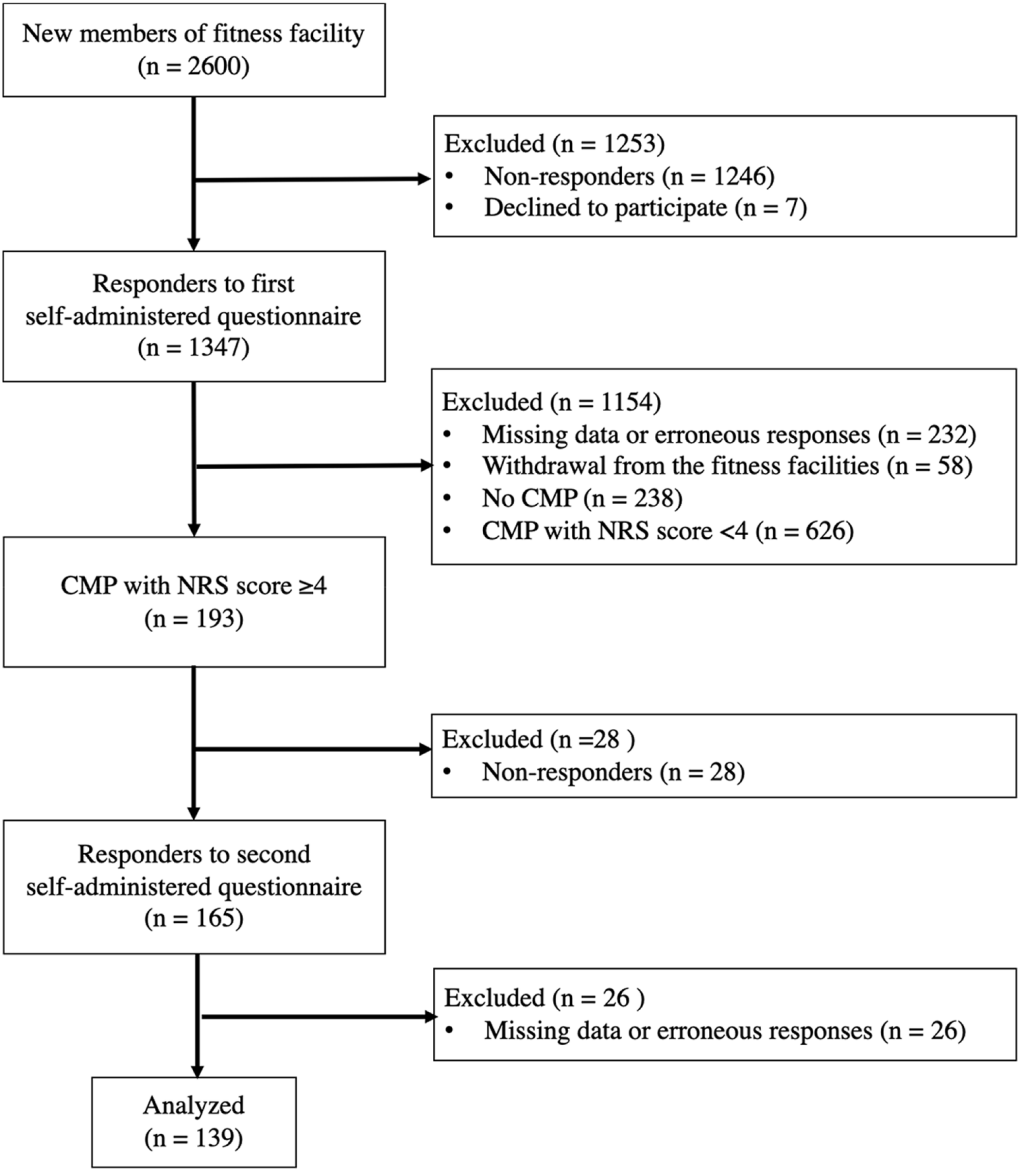
Flow diagram of participant selection. CMP = chronic musculoskeletal pain , NRS = numeric rating scale.

Participants were adult women who became new members of fitness facilities and performed circuit training for 3 months (“the intervention”) without any restrictions on exercise frequency. A total of 139 women with CMP who completed a set of questionnaires, including the numeric rating scale (NRS) for CMP, pain catastrophizing scale (PCS), Roland-Morris disability questionnaire (RDQ), Shoulder36, and Knee injury and Osteoarthritis Outcome Score (KOOS), before and 3 months after the intervention (Fig. 1). The baseline characteristics of the participants are presented in Table 1. The mean age was 63.1 years (SD = 10.8), and the mean NRS score was 5.3 (SD = 1.5).

**Table 1:** Participant baseline characteristics (n = 139).

Characteristics	
Age (years), mean (SD)	63.1 (10.8)
BMI (kg/m^2^), mean (SD)	24.5 (3.9)
Smoking, yes/no (%)	7.9
Analgesics, yes/no (%)	35.3
Conventional exercise, yes/no (%)	25.2
Urban, yes/no (%)	53.2
Number of pain site (score 0–35), mean (SD)	4.2 (2.8)
NRS (score 0–10), mean (SD)	5.3 (1.5)

BMI: body mass index; NRS: numeric rating scale; SD: standard deviation.

### Effects of exercise on pain and associated factors

We analyzed the change in pain after the intervention, as well as psychological and physical assessments (Table 2). Significant improvements were noted in the mean NRS score and number of pain sites after the intervention relative to baseline (p < 0.0001 and p = 0.0024, respectively). The prevalence of pain in the lower back and shoulder were significantly decreased (p = 0.0196 and 0.0280, respectively). With regard to pain catastrophizing, rumination, magnification, helplessness, and total PCS score were significantly improved after the intervention relative to baseline (p = 0.0017, 0.0047, 0.0387, and 0.0013, respectively). Physical function also improved after circuit training, as reflected in the RDQ and KOOS activities of daily living (ADL) scores (p = 0.0004 and 0.0295, respectively). In contrast, Shoulder36 scores did not change significantly after the intervention relative to the baseline. The achievement rates of minimal clinically important difference (MCID) were analyzed in the features with significant improvements such as NRS, PCS Total, RDQ, and KOOS ADL, each of which was based on past reports of satisfactory outcomes, as follows: NRS reduction of 1 point^32^, PCS reduction of 38%^33^ RDQ reduction of 2 points^34^, and KOOS ADL increase of more than 8^35^. Meanwhile, the MCID for NRS was achieved in 60.4% of the participants, and those for PCS, RDQ, and KOOS ADL were achieved in 35.3%, 30.9%, and 18.0% of the participants, respectively.

**T﻿able 2:**
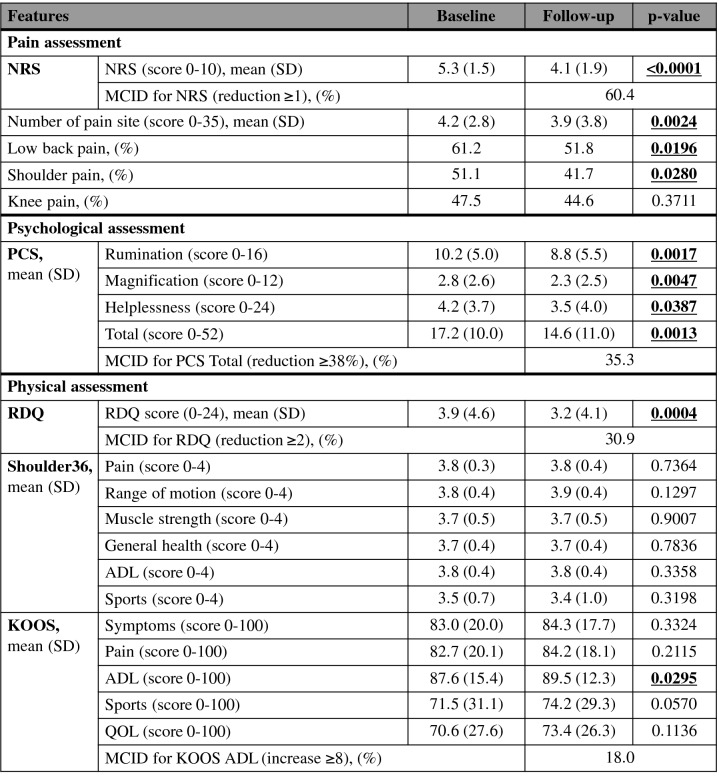
Comparison of pain and associated functions between baseline and 3 months after intervention.

NRS score, number of pain site, and the prevalence of pain in the low back and shoulder were significantly reduced after the intervention relative to baseline. In the psychological assessment, all PCS subdomain scores and the total score, were significantly improved after the intervention relative to baseline. For physical disability assessment, RDQ and the KOOS domain score for ADL were significantly increased, but Shoulder36 score did not significantly change, after the intervention relative to baseline. Group comparisons for continuous data were performed by Wilcoxon signed-rank test, and McNemar test was used for categorical variables.

NRS: numeric rating scale; PCS: pain catastrophizing scale; RDQ: Roland-Morris disability questionnaire; KOOS: knee injury and osteoarthritis outcomes survey; MCID: minimal clinically important difference; SD: standard deviation; ADL: activities of daily living.

Significant differences are indicated in bold.

### Effect of exercise dose on NRS, PCS, RDQ, Shoulder36, and KOOS scores

We further divided the participants into three groups according to exercise frequency: the bottom 33% of participants exercised ≤ 23 times for 3 months and were assigned to the low-dose group (n = 48); the middle 33% of participants exercised 24–33 times for 3 months and were assigned to the moderate-dose group (n = 46); and the top 33% of participants exercised ≥ 34 times for 3 months and were assigned to the high-dose group (n = 45) according to the previously reported studies^36,37^ with modification. We evaluated the effects of exercise on pain and its associated functions according to dose. The results are summarized in Table 3. NRS scores were significantly improved in all three groups after the intervention relative to baseline (p = 0.0008, 0.0005, and 0.0002, respectively). The total score, as well as rumination and magnification domain scores, of PCS were significantly lower in the high-dose group after the intervention (p = 0.0091, 0.0300, and 0.0196, respectively). The total score and rumination domain score also improved in the moderate-dose group (p = 0.0190 and p = 0.0258, respectively). However, no improvement was observed in the low-dose group. Significant improvements were observed in RDQ scores for the moderate-and high-dose groups after the intervention relative to baseline (p = 0.0123 and 0.0009, respectively), but no significant change was observed in the low-dose group. Next, we evaluated the change in the Shoulder36 and KOOS scores depending on exercise frequency. None of the Shoulder36 domain scores differed significantly between the groups. With regard to KOOS scores, symptoms, sports, and QOL domain scores were significantly improved in the moderate-dose group (p = 0.0159, 0.0061, and 0.0423, respectively), and pain and ADL domain scores were significantly improved in the high-dose group (p = 0.0236 and 0.0046, respectively), whereas significant changes were not observed in the low-dose group.

**Table 3:**
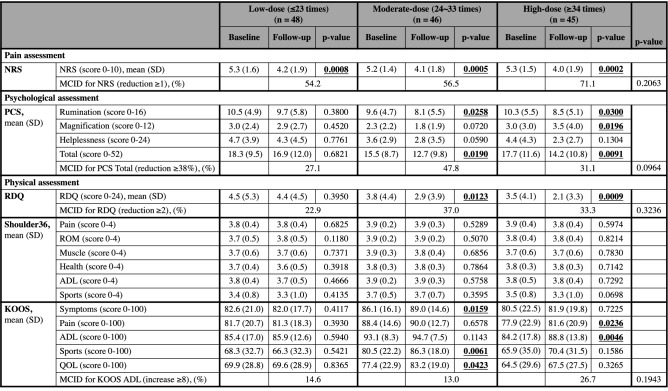
Comparison of pain and associated functions in each groups between baseline and 3 months after intervention using Wilcoxon signed-rank test.

NRS scores were significantly improved in all groups after the intervention relative to baseline. In the Moderate-dose and High-dose groups, PCS, RDQ, and KOOS scores were significantly improved after the intervention relative to baseline, however, significant differences were not found in the Low-dose group. Shoulder36 scores did not significantly change after the intervention relative to baseline in any of the groups. Fisher’s exact test was used to evaluate intergroup differences of MCID achievement, and there was no significant difference among the groups.

NRS: numeric rating scale; PCS: pain catastrophizing scale; RDQ: Roland-Morris disability questionnaire; KOOS: knee injury and osteoarthritis outcomes survey; MCID: minimal clinically important difference; SD: standard deviation; ADL: activities of daily living.

Significant differences are indicated in bold.

Collectively, these results suggest that NRS scores were significantly improved regardless of exercise frequency, and PCS, RDQ, and KOOS domain scores were significantly improved in the moderate- and high-dose groups, but not in the low-dose group.

We also investigated intergroup differences in the achievement rates of the MCID. In all groups, more than half of the participants achieved MCID for the NRS, especially in the high-dose group who had an achievement rate of 71.1%. However, there was no significant difference compared to the other two groups. Regarding the other MCIDs, the achievement rate of the low-dose group tended to be low in each MCID; however, no significant difference was observed.

### Odds ratios (ORs) for withdrawal from the fitness facilities after the 3-month follow-up

**Figure 2:**
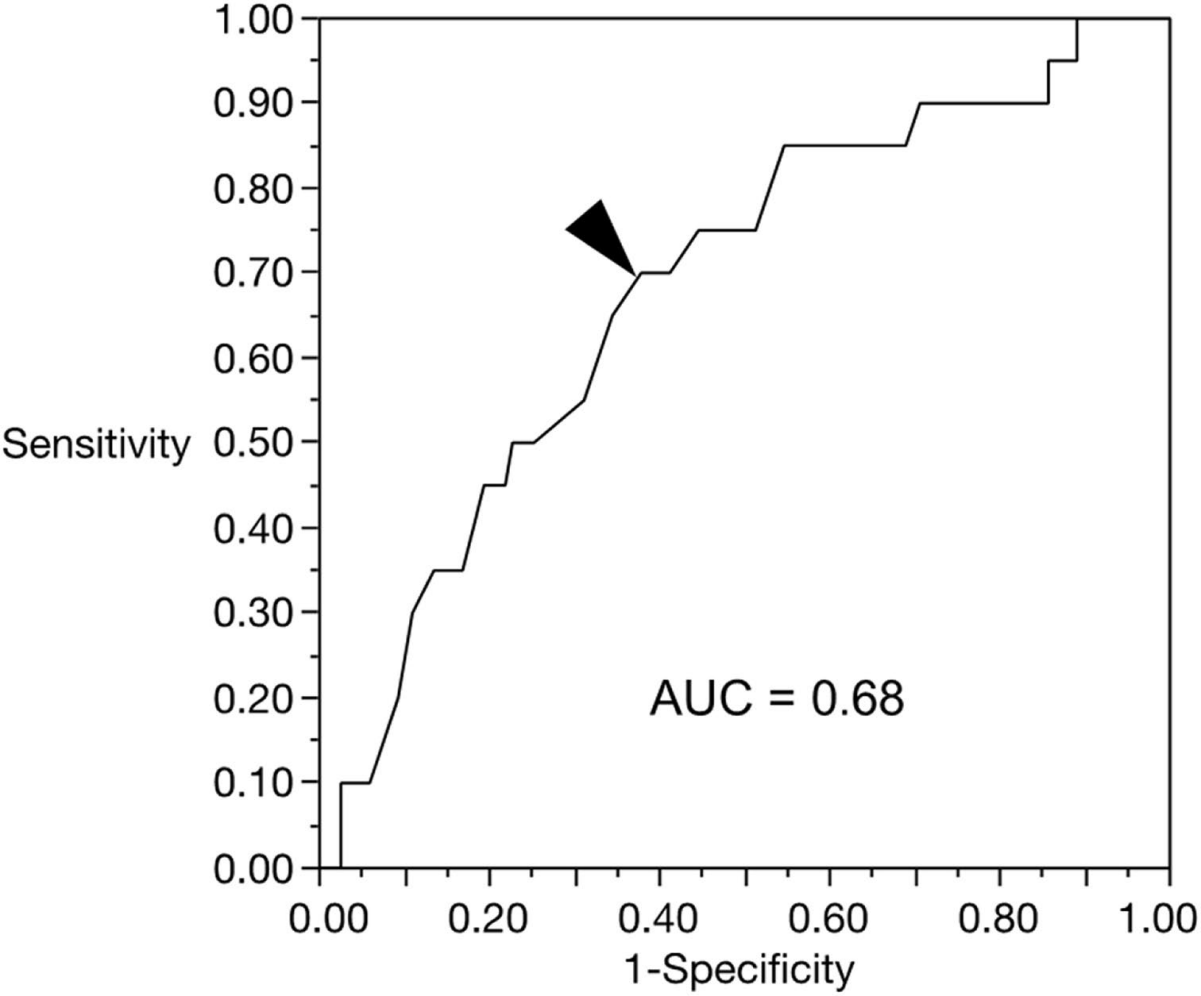
ROC curve for the exercise frequency as a predictor of drop-out (AUC = 0.68). The triangle on curve shows optimal cut-off point (25), corresponding with the maximum sum of sensitivity (70.0%) and specificity (62.2%). ROC = receiver operating characteristic, AUC = area under the curve.

The results obtained thus far suggest that continuous exercise is important for improving CMP and associated functions; therefore, we further investigated the predictors of dropout. Within 3 months of the study period, 20 people withdrew from the fitness facilities. Table 4 shows the multivariable logistic regression model for dropout after the 3-month follow-up, and the exercise frequency was associated with reduced odds of dropout (OR: 0.94 [95% CI 0.90–0.99], p = 0.0127). We further evaluated the threshold levels associated with dropout using the receiver operating characteristic (ROC) method. The appropriate cut-off point for the exercise frequency was 25 sessions over the course of 3 months, with a specificity of 62.2% and a sensitivity of 70.0% (Fig. 2). The area under the ROC curve (AUC) was 0.68. This suggests that exercising at least 25 times in 3 months, which was equivalent to exercise twice a week, was a predictor of preventing dropout of the circuit training.

**Table 4:**
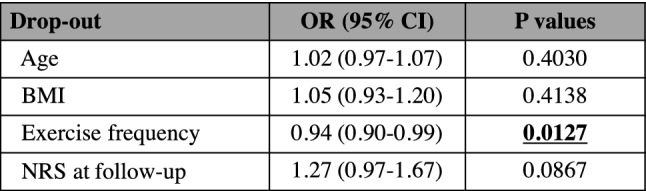
Odds ratios for drop-out of the fitness facilities after the 3-month follow-up (n = 20).

Exercise frequency was significantly associated with the drop-out (p = 0.0127).

BMI: body mass index; NRS: numeric rating scale; OR: odds ratio; CI: confidence interval.

Significant differences are indicated in bold.

## Discussion

To the best of our knowledge, this study is the first to assess the effects of an aerobic-resistance circuit training regimen on pain, pain catastrophizing, and physical function in adult women with CMP. The main findings of this study indicate that CMP, psychological vulnerability, and physical function improved after 3 months of circuit training in Japanese women.

To investigate the effect of exercise on pain and its associated factors, we focused on the low back, shoulder, and knee in this study, given the high prevalence of CMP at these sites^3,38^. We also used MCID as a satisfactory outcome to assess correlations between exercise frequency and improvement in pain-associated factors. MCID was first described by Jaeschle et al.^39^ as “the smallest difference in score in the domain of interest which patients perceive as beneficial and which would mandate, in the absence of troublesome side effects and excessive cost, a change in the patient’s management.” It is a useful benchmark for determining whether patients show sufficient improvement to notice a real clinical difference. In this study, the participants showed a significant improvement in the mean NRS score beyond the MCID. Some studies have reported that combined exercise improves low back pain and knee function. For instance, in one study, a general rehabilitation program combining strength, stretching, endurance, balance, and stabilization exercises in patients with chronic low back pain had significant effects on pain and disability^40^. A systematic review concluded that combined exercises are effective for improving functional limitations resulting from knee osteoarthritis^41^. Similar to these reports, a significant reduction in low back pain and improvement in low back and knee function were also observed in the present study. Previous reports on exercise for women with CMP showed that the combined training of neuromuscular exercise and back care counseling was effective for recurrent non-specific low back pain^42^, and resistance training was also effective for chronic neck pain^43^. In this study, it was predicted that circuit training would positively affect women with CMP. The prevalence of shoulder pain was also significantly decreased after the intervention relative to baseline, but no significant improvement was observed in shoulder function. This might be explained by the high baseline score for shoulder function, suggesting that many participants in this study had shoulder pain but not shoulder disability. According to Dunn et al.^44^, shoulder pain is not related to cuff tear severity, but might be correlated with comorbidities, lower education level, and ethnicity. This previous report may explain the high shoulder score of Shoulder36 in this study.

In this study, the effects of the exercise dose on the NRS, PCS, RDQ, Shoulder36, and KOOS scores were also examined. Although scores of PCS, RDQ, and KOOS significantly decreased in the moderate-and high-dose groups, these effects were not observed in the low-dose group. Furthermore, participants who exercised less than twice a week were less likely to achieve MCID for pain and its associated factors. This suggests that circuit training was more effective in participants who exercised more than 24 times in a 3-month period than in those who exercised less. Participating in training 24 times in 3 months is equivalent to exercise twice a week. Our findings collectively indicate that aerobic-resistance circuit training in adult women with CMP had a significant association with reducing pain, and continuous exercise for at least twice a week is required to improve psychological vulnerability and physical disability. Interestingly, there was no significant change in the shoulder function. There is a lack of studies in the literature on the impact of exercise dose on pain^14^, and the American College of Sports Medicine (ACSM) does not have a specific exercise recommendation to address pain. The ACSM’s Position Stand recommends 30 min of moderate-intensity walking exercise for 5 days per week (or 150 MET minutes) to sufficiently sustain adequate cardiorespiratory, musculoskeletal, and neuromotor fitness for healthy adults^45^. Although long-term adherence to this regimen would be ideal, it is likely too high of a starting point for individuals with chronic pain, especially for those suffering from movement-induced pain and movement-associated fear avoidance behavior^46^. Therefore, it is important to carefully examine the optimal exercise doses to reduce pain. Given that previous studies reported that pain can be reduced by exercising twice^47^ or 3 times per week^48^, we divided participants into three groups based on exercise frequency. The participants were divided into two groups: less than twice (low-dose group), more than twice and slightly less than 3 times (moderate-dose group), and more than 3 times per week (high-dose group). Circuit training was more effective in the moderate-and high-dose groups than in the low-dose group, and 30 min of continuous exercise for at least twice a week was found to be associated with improvement in both CMP and physical function.

In the results of OR for withdrawal from the fitness facilities after the 3-month follow-up, exercise frequency was significantly associated with dropout from the fitness facilities. Furthermore, the cutoff point was 25 times greater in the 3-month period, equivalent to twice a week. In a randomized comparative trial of combined training for adults with chronic nonspecific low back pain, participants exercised twice a week for 1.5 h^49^. This contrasts with the present study, in which a sufficient effect was obtained even with 30-min exercise sessions twice a week. This result suggested the time efficiency of the circuit program, as shown in the comparison with the combined program in a previous report^25^. In addition, as reported previously, people with CMP appear responsive to lower exercise dosage. These are important indications, given that “lack of time’ is the primary reason people do not exercise^50^. Since adherence to circuit training was the key predictor of improved CMP and prevention of dropout in the results of this study, future efforts should focus on strategies to improve adherence. Exercise programs that integrate both resistance exercise and aerobic training have increased in demand because of their ability to meet exercise guidelines in a time-efficient manner^51^. Moreover, circuit training allows numerous people to participate in the same training session because of the low total duration of the exercises^24,25^, which promotes high retention and adherence^52^. Exercising with others has also been reported to be useful for preventing depression in older adults^53^. In addition, the safety of aerobic-resistance circuit training has been demonstrated in men with post-myocardial infarction^26^. Taken together, circuit training is sufficiently safe and may reduce the dropout rate by promoting exercise with other participants without requiring major time commitment.

Patients with CMP complain of widespread negative consequences, such as significant pain intensity, depressive symptoms, weakness, sleep-related problems, sick leave, loss of enjoyment of life in general, and decreased emotional well-being^54^. Physiological pain with organic insults can have negative effects on emotions and cognitive function, and conversely, a negative emotional state can lead to increased pain through the central pain pathway^55^. Several theoretical frameworks on pain catastrophizing and pain-related outcomes have been proposed^56,57^. A multidisciplinary approach that included exercise therapy, psychotherapy, and patient education reportedly improved pain, PCS, and physical function in patients with CMP^7^. Though the result was similar to the current study, the difference is whether the place of treatment was a hospital or a fitness club. A multidisciplinary approach requires a large number of medical staff, including orthopedic surgeons, psychiatrists, nurses, physical therapists, clinical psychologists, pharmacists, and nutritionists, and it was effective for patients who visited the hospital for pain treatment. For subjects motivated to exercise, such as fitness club members, circuit training seems to be effective and has an advantage in terms of medical costs, compared with a multidisciplinary approach. CMP is a substantial medical health issue, and the financial costs to society are enormous, with current estimates running at more than €200 billion per annum in Europe and $150 billion per annum in the United States^58^. According to research performed by the American Chamber of Commerce in Japan (ACCJ) in 2011, estimated economic losses due to pain were ¥35 million (approximately $340 thousand) a year, excluding treatment costs^2^. Therefore, exercise is important not only for improving CMP and associated functions, but also for improving quality of life and healthcare costs. In the present study, 35.3% of all patients with CMP with NRS scores ≥ 4 used oral analgesics. Although the proportion of oral analgesic use did not significantly differ by exercise frequency, there was a tendency for higher oral analgesic use with lower exercise frequency (data not shown). Continuous exercise may help reduce medical expenses, and fitness programs that incorporate both aerobic fitness and strength training would cost little to implement in terms of equipment and facilities^59^.

The present study has several limitations. First, our sample population consisted of only adult females and was mostly Japanese. Future studies should examine whether our results are generalizable to men and people of other ages and races. The average age of the participants in the current study was 63.1 years. In this regard, older age has been reported as a predictor of poor outcomes in patients with neck pain treated by physical therapy^60^. Furthermore, African-Americans with chronic pain reportedly have different rehabilitation outcomes than Caucasians, and Asians have higher pain catastrophizing and pain severity than European Canadians^61^. Second, a limitation of the present study was the variation in exercise frequency in each group. In the low-dose group, exercise frequency varied from 3 to 23 times. In addition, due to the nature of the observational study, it was not possible to include a control group (e.g., without intervention). Third, all outcomes were based on subjective evaluations from questionnaires and were not objectively evaluated. As such, evaluations of muscle strength, physical performance, and pressure pain threshold are necessary in the future. Fourth, our inclusion and exclusion criteria and screening methods may have impacted the external validity of our findings. Some potentially important demographic variables (i.e., education, income, and employment status) were not surveyed. Population-based studies of chronic pain have consistently shown that chronic pain occurrence is inversely related to the socioeconomic status, with evidence that people living in adverse socioeconomic circumstances experience more chronic pain and greater pain severity, independent of other demographic and clinical factors^5^. In this study, all the participants were fitness club members and were presumed to have a favorable socio-economic status. Finally, the follow-up period was relatively short. More studies with longer follow-up periods are needed to evaluate long-term outcomes.

In conclusion, CMP, pain catastrophizing, and physical function in adult female fitness club participants with a CMP of NRS 4 or higher improved after 3 months of aerobic-resistance circuit training. The subjects included 139 out of 2600 new fitness club members, with an average age of 63.1 years. It was suggested that circuit training has the potential for immediate and high clinical impact as a low-cost, non-pharmacological, and non-invasive treatment.

## Methods

### Participants

The structured combined exercise training facilities (Curves Japan Co., Ltd.), to which the participants of the study belonged, were fitness facilities that held 30-min circuit training sessions for women in Japan. Participants of the present cohort study were women who became members of the above-mentioned facilities. The first self-administered questionnaire regarding medical history and pain-associated functions was distributed at the time of enrollment to 2600 members of 132 facilities in Japan, and responders were 1347 people.

The inclusion criteria were participants who have CMP with an NRS score of 4 or higher. The question related to the presence of CMP was “Please show all parts of the body that have experienced persistent or recurrent pain over the last 3 months or more on the image below.” Regarding the answers, participants were asked to “check the painful parts of the body” or indicate “no pain,” based on the health condition at the time of answering the questionnaire. The Michigan Body Map^62^, a self-report measure to assess areas of the body with chronic pain, was used to assess the location of pain and number. Multiple answers were allowed for the location of pain. Chronic pain was defined as pain that persisted for more than 3 months, based on a previous report^6^. Participants were asked the NRS score of the single most painful site. The exclusion criteria were as follows: missing data for height, weight, age, CMP, NRS, exercise frequency, errors in these measurements, or clear entry errors (n = 232), withdrawal from the fitness facilities (n = 58), absence of CMP on enrollment based on responses to the self-administered questionnaire (n = 238); and CMP with NRS score < 4 (n = 626). The second self-administered questionnaire was administered to 193 members 3 months after admission. Consequently, 165 people responded to the second self-administered questionnaires, and 139 members, excluding those with missing data, were included in the present study. Pain with an NRS score ≥ 4 is subject to drug treatment^63^ and categorized as moderate or severe pain^64^. Accordingly, we defined participants with an NRS score of ≥ 4 as having CMP in need of treatment (Fig. 1).

### Study design

This was a multicenter cohort study that examined the effects of aerobic-resistance circuit training for 3 months in women with CMP. All procedures were approved by the ethics committee of Osaka University. Written informed consent was obtained from all the participants prior to their participation. All primary and secondary pre-specified efficacy outcomes are reported in the present manuscript, and all methods were performed in accordance with the relevant guidelines and regulations.

### Exercise regimen

The number of admissions to fitness facilities was examined for the first 3 months after joining using an electronic admission management system. Since the number of admissions corresponds to the number of training sessions attended, the former was defined as the exercise frequency. To perform the aerobic-resistance circuit training, 12 individual exercise stations designed to work on all major muscle groups and 12 step boards were arranged alternatively in a circular manner. The circuit exercise alternated between 30 s of resistance exercise and 30 s of aerobic training, leading to a duration of 24 min. Combined with the 6-min stretching period, the training lasted for 30 min. The exercise involved stepping on a step board for the aerobic training while executing resistance training using 12 different hydraulic devices (chest press/seated row, squat, shoulder press/lateral pull, leg extension/leg curl, abdominal crunch/back extension, lateral lift, elbow flexion/extension, horizontal leg press, pectoral deck, oblique, hip abductor/adductor, gluteus), and was developed for women to increase their muscle strength. All participants performed both aerobic and resistance training in the same manner, allowing numerous people to participate in the same training session because of the short total duration of the exercises.

### Outcomes

All participants completed validated questionnaires pertaining to their general health, physical activity levels, and physical function, which included 34 items (medical history, pain site, pain intensity, pain duration, pain frequency, current treatment of pain, smoking, reason for joining the fitness club, etc.) other than PCS, RDQ, Shoulder36, and KOOS. The participants took approximately 30 min to complete the questionnaire. Outcomes included changes in pain and associated factors, as follows: intensity of pain as assessed by NRS (0: no pain, 10: unbearable pain), number of pain sites, prevalence of chronic pain, pain catastrophizing as assessed by PCS (0: low level of catastrophic thinking, 52: high level of catastrophic thinking), low back pain-specific disability as assessed by RDQ (0: no limitation, 24: maximal limitation), shoulder function as assessed by Shoulder36 (0: maximal limitation, 4: no limitation), and knee function as assessed by KOOS (0: maximal limitation, 100: no limitation).

The NRS measures pain severity by asking the patient to select a number, and is considered one of the best single-item methods available to estimate pain intensity^65^.

Several assessment tools have been developed to quantify pain catastrophizing, the most commonly used of which is the PCS^66^. The PCS includes 13 items rated on a 5-point scale ranging from 0 (not at all true) to 4 (very true).” The items are divided across three subdomains: rumination (4 items, e.g., “When I have pain, I can’t keep it out of my mind”), magnification (3 items, e.g., “When I have pain, I keep thinking of other painful events”), and helplessness (6 items, e.g., “When I have pain, I feel like I can’t go on”).Items are summed across subdomains to derive a total score ranging from 0 to 52, with higher scores reflecting higher levels of catastrophic thinking^67^. Pain catastrophizing is the tendency to focus on and amplify pain sensations and feel helpless when pain occurs^59^.

The RDQ was designed in 1983^68^ for use in primary care research to assess physical disability due to low back pain, and it is sensitive to changes in patients with mild to moderate disability^69^. The Japan Orthopaedic Association and the Japanese Shoulder Society jointly developed an original patient-reported outcome measure, Shoulder36, version 1.3, in 2010^70^. Shoulder36 is a self-reported shoulder-specific instrument that evaluates shoulder function and consists of 36 questions that represent six domains: pain, range of motion (ROM), muscle strength, general health, activities of daily living (ADL), and ability of sports (Sports). Scores range from 0 to 4 in each domain, and summing the scores to obtain a total score is not permitted because the domains are not independent^71^. A strong association has been reported between the Simple Shoulder test and each domain in Shoulder36 in patients with rotator cuff tears, suggesting the usefulness of Shoulder36 as a patient-based scoring system in these patients^72^.

The KOOS is a 42-item joint-specific measure that was developed to assess short- and long-term outcomes in patients with knee injuries, as they are often at risk of developing knee osteoarthritis^73^. It consists of five domains that are scored separately from 0 (extreme problems) to 100 (no problems): symptoms, pain, activities of daily living (ADL), sport and recreation function (Sports), and knee-related quality of life (QOL).

### Statistical analysis

All recorded data were entered into the electronic storage. Statistical analyses were performed using the JMP Pro 15 software (SAS Institute Inc., NC, USA). Normality of the data was assessed, and statistical significance was set at p < 0.05.

Categorical variables are presented as frequencies and percentages. Quantitative variables are presented as mean (SD). Outcomes were defined as changes between 3 months after intervention and baseline for NRS, number of pain sites, prevalence of CMP, PCS, RDQ, Shoulder36, and KOOS. Comparisons were performed between the baseline and 3 months after the intervention. Because most parameters were not normally distributed, continuous data were analyzed using the Wilcoxon signed-rank test. The McNemar test was used to analyze categorical variables. Fisher’s exact test was used to evaluate intergroup differences in MCID achievement.

Multivariable logistic regression analysis was performed to calculate the ORs for dropout of fitness facilities. The dependent variable was dropout (0 = no dropout, 1 = dropout) at 3 months after the 3-month follow-up period. The selection of potential predictor variables for that particular regression model was based on previous literature, including age and BMI as the cause of dropout^74^. The variables that showed a significant univariate association with dropping out (p < 0.1), such as exercise frequency and NRS at follow-up, were also selected as potential predictor variables. The inclusion of appropriate predictor variables in the final multivariate regression model was based on multicollinearity assessment and the strength of their univariate association with the primary outcome measure. In the final multivariate logistic regression model for dropout status, statistical significance was set at p < 0.05. The ROC method was used to determine the threshold levels associated with dropout. This method has the advantage of synthesizing information on the sensitivity and specificity for detecting improvement using an external criterion^75^. From the ROC curves, we computed the optimal cut-off point, corresponding to the maximum sum of sensitivity and specificity.
